# High prevalence of chronic kidney disease of unknown etiology among workers in the Mesoamerican Nephropathy Occupational Study

**DOI:** 10.1186/s12882-022-02861-0

**Published:** 2022-07-07

**Authors:** Sinead A. Keogh, Jessica H. Leibler, Caryn M. Sennett Decker, Juan Jose Amador Velázquez, Emmanuel R. Jarquin, Damaris Lopez-Pilarte, Ramon Garcia-Trabanino, Iris S. Delgado, Zoe E. Petropoulos, David J. Friedman, Magaly Rosario Amador Sánchez, Raul Guevara, Michael D. McClean, Daniel R. Brooks, Madeleine K. Scammell

**Affiliations:** 1 Department of Environmental Health, Boston University School of Public Health, 715 Albany St. T4W, Boston, MA 02118 USA; 2 Department of Epidemiology, Boston University School of Public Health, 715 Albany St. T3E, Boston, MA USA; 3 Agencia para el Desarrollo y la Salud Agropecuaria (AGDYSA), San Salvador, El Salvador; 4 Centro de Hemodiálisis, San Salvador, El Salvador; 5 Emergency Social Fund for Health, Tierra Blanca, El Salvador; 6 Division of Nephrology, Harvard Medical School and Beth Israel Deaconess Medical Center, Boston, MA USA

**Keywords:** CKD, Chronic kidney disease of unknown etiology, Mesoamerican nephropathy, Occupational epidemiology, Workers, Cohort study, Kidney function, KDIGO, CKD-EPI, Prevalence

## Abstract

**Background:**

Mortality from chronic kidney disease of unknown etiology (CKDu) is extremely high along the Pacific coast of Central America, particularly among sugarcane workers. The Mesoamerican Nephropathy Occupational Study (MANOS) is a prospective cohort study of CKDu among agricultural and non-agricultural workers in El Salvador and Nicaragua. The objective of this manuscript is to describe the MANOS cohort recruitment, baseline data collection, and CKDu prevalence after two rounds.

**Methods:**

Workers with no known diabetes, hypertension, or CKD were recruited from sugarcane, corn, plantain, brickmaking, and road construction industries (*n* = 569). Investigators administered questionnaires, collected biological samples, and observed workers for three consecutive workdays at the worksite. Serum specimens were analyzed for kidney function parameters, and used to calculate estimated glomerular filtration rate (eGFR). At six months, serum was collected again prior to the work shift. CKD at baseline is defined as eGFR ≤ 60 ml/min/1.73m^2^ at both timepoints. Age-standardized prevalence was calculated by industry, country, and demographic measures. Kidney function parameters were compared by CKD status.

**Results:**

Prevalence of CKD at baseline was 7.4% (*n* = 42). Age-standardized prevalence was highest in Salvadoran sugarcane (14.1%), followed by Salvadoran corn (11.6%), and Nicaraguan brickmaking (8.1%). Nicaraguan sugarcane had the lowest prevalence, likely due to kidney function screenings prior to employment.

**Conclusion:**

Despite efforts to enroll participants without CKD, our identification of prevalent CKD among agricultural and non-agricultural workers in the MANOS cohort indicates notable kidney disease in the region, particularly among sugarcane workers.

## Background

Reported mortality rates from chronic kidney disease (CKD) in Central America have been increasing over the past three decades, with the highest rates (60–70 deaths / 100,000 population) among men in El Salvador and Nicaragua respectively [1, 2]. While CKD in the U.S. and Europe typically affects older men and women with near equal frequency, in Central America one form of CKD disproportionately affects younger men (< 50 years of age) who are manual workers along the Pacific coast [3, 4]. Widely referred to as Mesoamerican Nephropathy (MeN), or CKD of unknown etiology (CKDu), the disease is not associated with known CKD risk factors such as age > 60 years, diabetes, hypertension, glomerulonephritis or obesity [2–6]. Agricultural workers appear to be highly affected, suggesting the potential role of occupational exposures including agrichemicals and heat stress [5, 7]. Several studies have identified the disease among sugarcane workers [5, 7, 8].

Studies have also documented a high prevalence of low kidney function, suggestive of CKD, in non-agricultural workers including miners and construction workers [9, 10] but few have confirmed CKD based on two eGFR values as per clinical guidelines [11–13]. CKDu with similar characteristics is present among agricultural workers in other tropical regions including Sri Lanka and India [5, 7]. The extreme burden placed on affected families, communities, and healthcare systems has created an urgent public health problem [14].

While there are differing hypotheses about the primary causes, many CKDu researchers agree that the disease is likely multifactorial [4, 5, 7, 15, 16]. Frequently-cited risk factors include the individual and/or combined effects of 1) heat stress (including dehydration), 2) exposure to agrichemicals, heavy metals, or infectious agents, 3) the use of nephrotoxic medications, such as non-steroidal anti-inflammatory drugs (NSAIDs), 4) muscle injury, and 5) genetic susceptibility [4, 5, 7, 16]. The combination of heat stress and dehydration is viewed by many investigators as a likely contributor to CKDu, with the proposed mechanism of repeated episodes of clinical or sub-clinical acute kidney injury (AKI) leading to kidney function decline [17–19]. Nearly all evidence collected to-date would support the assertion that CKDu is an occupational illness [8, 20].

Nephrotoxicants such as agrichemicals and heavy metals are of significant interest but very few studies thoroughly characterize exposures, either alone or in combination [5, 7, 21, 22]. Glyphosate is a widely used herbicide in Central America and has been demonstrated to cause kidney injury in rats; the basis for the U.S. Environmental Protection Agency’s reference dose [23]. The relationship between glyphosate exposure and kidney toxicity among humans is less clear [21, 24].

Several heavy metals are associated with kidney damage at high levels or following short-term/high-dose exposure (e.g. arsenic, cadmium, lead, mercury and uranium). Until recently, no studies examined the nephrotoxic effects in humans following low-level chronic exposures, and none have examined this relationship prospectively among workers in the region [21]. Smpokou et al. conducted a community-based study in Nicaragua assessing baseline exposures and found high concentrations of total arsenic and tin, but did not observe an association with decline in kidney function, over the two-year study period [24].

The Boston University CKD Research Group has been studying CKDu in Central America since 2009 [25]. In 2017, we received an award from the National Institute of Environmental Health Sciences of the National Institutes of Health to initiate the Mesoamerican Nephropathy Occupational Study (MANOS), a study of agricultural and non-agricultural male workers in El Salvador and Nicaragua. The goals of MANOS are to: investigate the role of occupational and environmental exposures (including heat stress and dehydration, glyphosate, and heavy metals) on kidney injury and the decline of kidney function over time; communicate results to study participants; and collect and store biological specimens for testing future hypotheses. Due to its longitudinal design, MANOS permits us to assess CKD prevalence according to international clinical guidelines and follow workers prospectively.

This paper describes: 1) the design, planning and preparation for MANOS, 2) recruitment of the MANOS cohort, 3) baseline data collection, 4) baseline CKD prevalence, and 5) plans for cohort follow-up and future analyses.

## Methods

### Study design, planning and preparation

MANOS is a two-country longitudinal cohort designed to collect detailed occupational exposure information over three consecutive days at the worksite at baseline (Round 1) and follow-up with participants approximately every six months for two and a half years. Each follow-up includes the collection of biological samples (urine, whole blood, and serum) and questionnaire data (demographic, behavioral, and health-related information). Research team members are located in the United States, El Salvador and Nicaragua.

MANOS protocols were approved by the Boston University Medical Campus Institutional Review Board, the Salvadoran National Ethics Committee for Health Research, and two Nicaraguan review committees within the Nicaraguan Ministry of Health: The National Ethics Committee and the Office of Teaching and Research that oversees protocol for public health investigations.

To represent populations most affected by the disease, we focused MANOS recruitment on workers from the Pacific coastal regions of El Salvador and Nicaragua. The climate on the Pacific coast is tropical, with high temperatures year-round [26]. There are two seasons; dry (November–April) and wet (May–October), between which both agricultural and non-agricultural outdoor activities often vary. We hypothesized that seasonal weather and varied work activities during the year may affect exposures and kidney function [18]. We designed MANOS to alternate data collection between seasons, with Round 1 conducted during the dry season when the agricultural harvest occurs, and employment in many non-agricultural industries at its highest.

For several years prior to MANOS, our team of investigators from Boston University, Nicaragua and El Salvador, conducted research in the region, engaging communities and becoming familiar with industry practices and worker organizations, particularly the sugarcane industry in both countries [18, 27, 28], corn cooperatives in El Salvador [29] and artisanal brickmaking in Nicaragua [11]. We also engaged leaders in the medical and public health community in the region and in the US, including researchers at the US Centers for Disease Control and Prevention [30].

To enable comparison between multiple occupational settings with differing types and levels of exposures, we sought to include workers in agricultural and non-agricultural industries in each country ranging in size from small family businesses to cooperatives and multinational corporations.

All industries and companies were selected based upon reports of CKD, willingness of the industry/company leaders to cooperate, the feasibility of following workers over time based on where workers live in relation to the worksite, and geographic location along the Pacific lowlands. Among agricultural workers, we sought to recruit participants who were manual harvesters (among sugarcane also referred to as cane cutters) and who worked with pesticides. These are the jobs thought to have the highest heat and agrichemical exposures, respectively. Within sugarcane, we sought to capture potential differences in work practices and exposures; we included sugarcane workers in three companies in Nicaragua and two in El Salvador. The companies were selected to achieve variation in company structure and work practices (e.g., amount of sugarcane harvested, number of workers employed or contracted, job specialization, work shift start/stop times and duration) and geographic area within each country.

Among brickmakers in Nicaragua, we sought oven workers in addition to workers focused on other tasks, as our prior research indicated oven work as the job with the highest CKDu risk in the industry, hypothesized to be due to extreme heat exposure [11]. Road construction workers in El Salvador did not have such clear job delineations, nor did we have hypotheses regarding high versus low heat exposure jobs, and therefore we recruited generally across the workforce.

We drafted information about the study timeline, goals, and procedures to share with industry representatives as well as expectations regarding confidentiality of worker data and our commitment not to share individual results with employers, nor to publish the names of specific employers to protect the workers against possible stigma associated with our findings. MANOS investigators in each country worked with industry leaders on logistics of recruitment and data collection to avoid interfering with work practices or participant wages.

Nicaraguan and Salvadoran investigators led the MANOS field teams –medical doctors, nurses, and bioanalysts – in carrying out the study. In January 2018, simultaneous weeklong trainings were held with Boston University investigators in both countries to review protocol, equipment, and data management procedures. Teams communicated with each other via Skype and WhatsApp, sending photos, troubleshooting equipment, and fine-tuning the protocol.

### Participant recruitment

**Fig. 1:**
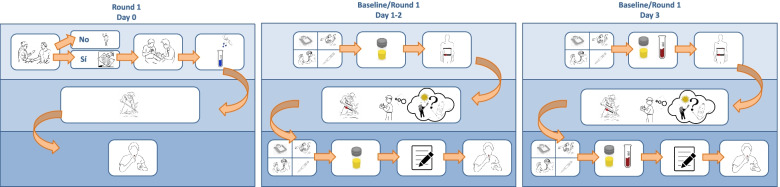
Infographics of MANOS Round 1 data collection process which were shared with workers.

Prior to recruitment, investigators held information sessions at each worksite providing workers with an overview of the study and an opportunity to ask questions. Posters, pamphlets and PowerPoint slides with infographics were shared to describe study activities (Fig. 1).

Recruitment occurred near the beginning of the workweek to increase the likelihood of collecting three consecutive days of data for each individual. On “Day 0,” the day of recruitment, MANOS field team members arrived early at the worksite to meet with the workers as they arrived, before the start of the workday. Participants were recruited in groups of up to 20 workers/week in each country. This number was feasible for our study team to recruit and monitor each day. Workers at each worksite would gather for the initial screening and recruitment. In sugarcane in both countries, workers tended to travel from field to field in groups of 30. At each of the other worksites, we focused our recruitment activities in areas where workers would be clustered, so we could monitor them more easily over the workday.

#### Eligibility criteria

Workers were screened to determine their eligibility. Only male workers age 18 to 45 years were included. Age is a risk factor for CKD and we wanted to recruit men who were least likely to have CKD due to age. Participants had to have worked in their current occupation for at least one season to increase our chances of being able to follow them at the workplace in the future, and so that measured exposures could be reasonable proxies of recent exposures in prior work seasons. We excluded workers with a prior diagnosis of CKD or other related health outcomes (e.g., diabetes, HIV, hepatitis B/C, and polycystic kidney disease) to limit potential confounders of the association between exposures and decline of kidney function over time. Workers who reported hypertension (a cause and consequence of kidney disease) were excluded only if they also reported current use of medication to control hypertension and/or a recent blood pressure > 160/95 mmHg. Finally, workers with contraindications for use of the CorTemp® Sensor pill, an internal body temperature sensor, were excluded: less than 80 pounds, obstructive disease of the gastrointestinal tract, impaired gag reflex, prior gastrointestinal surgery, hypomotility of the gastrointestinal tract, and having a pacemaker or other implanted electronic medical device.

Workers deemed eligible were consented by a member of the MANOS field team who read the consent form aloud. Within the consent form were five questions that gave participants the choice to “opt-in” to: 1) receive kidney function results; 2) receive results of metals analyses indicating health risk (i.e., high concentrations of metals for which there is a reference concentration according to U.S. public health agencies); 3) have urine, blood and saliva samples saved for future research on CKD; 4) have DNA stored for future analyses; and 5) be contacted for future studies. Participants received compensation in the form of cash payments for each round of data collection.

### Data collection

#### Round 1 questionnaire

**Fig. 2:**
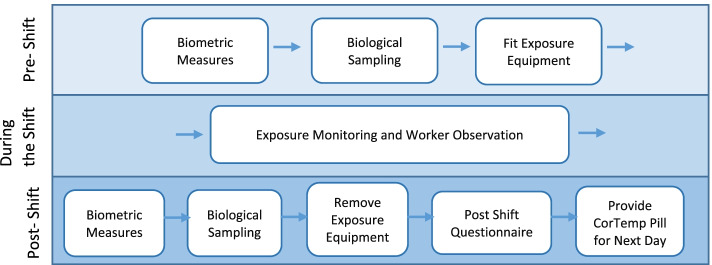
Round 1 data collection process at the work site.

After consenting workers, MANOS field team members administered a questionnaire on demographics, current and past occupation, work and home agrichemical use, personal protective equipment use, health and medication use, hydration practices, diet, alcohol and tobacco use, family history of CKD, and an alternative contact person. Day 0 questionnaires took approximately 45–60 minutes to administer. Days 1–3 of Round 1 data collection consisted of exposure monitoring during the work shift, pre- and post- shift physical examinations and biological sample collection, and a post-shift questionnaire (Figs. 1 and 2).

#### Physical examinations

Weight, blood pressure, and tympanic temperature for each participant were measured prior to and after each day’s work shift. Height was measured prior to the first day of monitoring.

#### Environmental heat monitoring

Waterless Wet Bulb Globe Thermometers (WBGT) measured ambient temperature, humidity, air flow, and radiant heat during the work shift. Devices were situated in work sites at locations as representative as possible of the microclimate of participants.

#### Personal monitoring

MANOS participants were fitted with devices to continuously monitor individual physical activity (accelerometer, right hip), heart rate (heart rate monitor, chest), and internal core body temperature (T_c_) throughout the work shift. CorTemp® Ingestible Core Body Temperature Sensors are vitamin pill-sized capsule that, once ingested, move through the body’s gastrointestinal tract and wirelessly transmit T_c_ readings to a receiver. The receiver was fitted in the small of the back with a nylon running belt and programmed to collect T_c_ data in 10-second intervals.

#### Biological sampling

Urine samples were collected before and after the work shift on all three days. Blood samples were collected before and after the work shift on Day 3 only. Every participant also provided a saliva sample to preserve for later genetic analyses.

#### Post-shift questionnaire

The post-shift questionnaire asked participants about that day’s work experience (i.e., intensity, schedule, and climate), hydration, medication use, symptoms, and use of personal protective equipment (PPE), and required approximately 30 minutes to administer.

#### Data transfer

In each country, data from personal monitoring devices were transferred daily to secure study computers. Data from questionnaires and data collection sheets were manually entered into Research Electronic Data Capture (REDCap) hosted at Boston University (CTSI 1UL1TR001430) [31].

#### Biological sample processing, analysis and report-Back

MANOS investigators in each country established an indoor biological sample processing site that was cool, clean, dust-free. Upon transfer of samples from the field, lab technicians analyzed urine for osmolality with a handheld refractometer and then for specific gravity, pH, leukocytes, nitrites, protein, glucose, ketones, urobilinogen, bilirubin, and blood with urinalysis dipsticks. Optical dipstick readers were used to improve the accuracy of results. Microscopic urinalyses were also conducted.

Biological samples were stored in a − 80 °C freezers at the Agency for Agricultural Development and Health (AGDYSA) in San Salvador and at the Ministry of Health National Laboratories (CNDR) in Managua. After the completion of baseline data collection, samples were sent by international courier on dry ice to Boston University Medical Campus (BUMC) to be shipped elsewhere for analysis or retained for storage. Saliva samples were stored at ambient temperature in closed collection kits inside 50 mL protector conical tubes and sent to Beth Israel Deaconess Medical Center in Boston, MA.

Our plan was to have serum creatinine measurements from both countries at all rounds analyzed at CNDR in Nicaragua. Due to political and social instability in Nicaragua, only the Round 1 samples from Nicaragua were analyzed at CNDR. Samples from El Salvador were shipped to BUMC for analysis at Quest Diagnostics. Serum creatinine (IDMS-traceable), calcium, chloride, glucose, phosphate (as phosphorus), potassium, sodium, urea/urea nitrogen, and uric acid were analyzed at both laboratories. Total creatinine phosphokinase was additionally analyzed at CNDR and albumin and carbon dioxide were additionally analyzed at Quest Diagnostics. A subsequent validation study indicated that serum creatinine values analyzed at CNDR were comparable to those analyzed at Quest Diagnostics. Samples from subsequent rounds from both El Salvador and Nicaragua were analyzed at Quest Diagnostics.

After laboratory analyses were completed, study clinicians provided each participant an individualized report with kidney function results from pre-shift serum analyses, basic urinalysis, and hemoglobin and hematocrit values with their respective reference ranges.

### Data analyses

Estimated glomerular filtration rate (eGFR) for each participant was calculated using the Chronic Kidney Disease Epidemiology Collaboration (CKD-EPI) equation for males of white or other ethnicities [32, 33]. CKD status for each participant was determined based on the two eGFRs separated by a period of at least three months recommended by the Kidney Disease Improving Global Outcomes (KDIGO) consortium [12]. Because proteinuria is not characteristic of MeN or CKDu [5], and we did not gather information about physical abnormalities of the kidney, we considered an individual to have CKDu if they presented a pre-shift eGFR < 60 mL/min/1.73m^2^ at both baseline (Round 1) and at a 6-month follow-up (Round 2), representing stages 3–5 CKD [12]. Participants with only Round 1 eGFR ≥60 mL/min/1.73m^2^ and no data in Round 2 were not considered as having CKD as per KDIGO guidelines requiring two measures of eGFR to determine disease status [12]. We assessed crude CKD prevalence by age group (18–24; 25–34; 35–45 years), family history of CKD (defined as having a father or brother with CKD), country, and industry. To minimize confounding by age, we calculated age-standardized prevalence measures using the method outlined by Rothman [34]. Age was considered categorically, and stratum-specific estimates were standardized to the age distribution across the study population. Age-standardized prevalence estimates were generated alongside 95% confidence intervals using Cochran’s formula [35].

Additionally, we used age-adjusted linear regression models to compare means between participants with and without CKD for each pre-shift serum kidney function parameter: albumin, calcium, carbon dioxide, chloride, creatinine, creatine phosphokinase, glucose, urea nitrogen, phosphorous, potassium, sodium and uric acid. Age was treated as a categorical measure. All analyses were conducted in Stata IC 16.1. The dstdize package was used to calculate age-standardized prevalence [36].

## Results

**Fig. 3:**
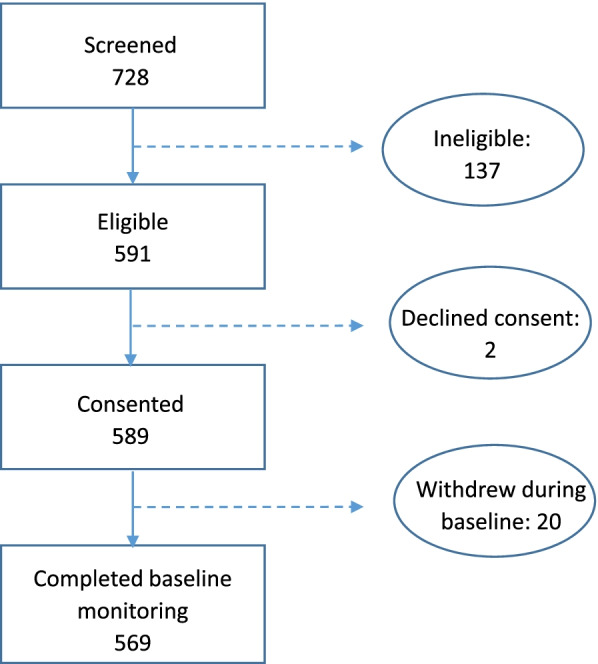
Cohort derivation.

Recruitment and Round 1 data collection took place between January and May 2018. Over 80% of workers screened were eligible to participate in the MANOS cohort (Fig. 3). Of the workers deemed ineligible (*n* = 137), most did not meet the age inclusion criteria of 18–45 years (*n* = 76; 56%) or had not worked at least one year/season in their current occupation (*n* = 38; 28%). Workers deemed ineligible due to preexisting conditions (*n* = 17; 12%) reported hypertension, polycystic kidney disease, renal insufficiency (a local synonym for CKD), diabetes, and hepatitis B or C. Six workers (4%) were excluded for having conditions contraindicated for CorTemp sensor use. Two eligible workers who were screened decided not to participate, resulting in 589 workers who gave their written, informed consent (Fig. 3).

During the consent process, all participants opted in to receive their kidney function laboratory results. All but one participant opted in to be contacted for future studies, and all but two gave permission for their biological samples to be saved for future research. The majority of participants (*n* = 572; 97%) gave permission for their DNA to be saved for future research. Of the 589 workers recruited, 569 (97%) completed Round 1 monitoring and constitute the MANOS cohort (Fig. 3).

### Participant characteristics

At baseline, MANOS cohort participants were fairly evenly distributed between El Salvador (*n* = 279) and Nicaragua (*n* = 290) and included workers in sugarcane, corn, plantain, brickmaking and road construction (Table 1). Almost three quarters of participants were agricultural workers (71%). By design, the highest percentage of participants were employed in sugarcane (41%), followed by corn (19%) and brickmaking (19%) (Table 1). Plantain and road construction workers each comprised approximately 10% of the cohort. Most participants reported no more than primary school as their highest level of educational attainment and the majority of participants reported living at their current residence for more than ten years.

**Table 1:** MANOS Cohort at Round 1

**Participants**	**N (%)**
MANOS Cohort	569 (100)
**Country**	**n (%)**
El Salvador	279 (49.0)
Nicaragua	290 (51.0)
**Age - years**	**n (%)**
18–24	172 (30.2)
25–34	250 (43.9)
35–45	147 (25.8)
**Education - Highest Level**	**n (%)**
No formal schooling	60 (10.5)
Primary	275 (48.3)
Secondary	219 (38.5)
University	15 (2.6)
**Years in Current Residence**	**n (%)**
> 10	374 (65.7)
6 to 10	83 (14.6)
2 to 5	81 (14.2)
< 2	30 (5.3)
Missing	1 (0.2)
**Industry**	**n (%)**
Sugarcane	235 (41.3)
Brick	107 (18.8)
Corn	110 (19.3)
Plantain	59 (10.4)
Road construction	58 (10.2)

Work activities over the three-days of Round 1 were more diverse in agriculture than anticipated (Table 2) and would change over the coming months and years. Plantain and sugarcane workers in Nicaragua and El Salvador respectively, engaged in more diverse activities than harvesting and pesticide application (Table 2). There was less job specialization at these sites than among sugarcane in Nicaragua. We made the decision to recruit the first 20 eligible workers at the work site on any given day, rather than restrict by job task. The field teams in each country arrived early to the fields of plantain and corn, and recruited participants as they arrived. In the case of corn, we received permission from the cooperative leaders to set up the study tents in the fields where they informed us harvesting would occur each week, until reaching our numeric recruitment goal. In the case of Brick, we went from work place to work place, sometimes dividing our team across several neighboring brick-making operations to recruit groups of 10 workers. Finally, road construction workers were all employed by one company which agreed to inform our team of their exact location during recruitment. As with agriculture, they recruited as workers arrived. Nearly all workers approached at each site agreed to participate.

**Table 2:** Primary job tasks in person-days over three days of monitoring at baseline by country, industry and company

	El Salvador				Nicaragua				
	SUGAR1	SUGAR2	CORN	CONS	SUGAR1	SUGAR2	SUGAR3	PLANT	BRICK
**Total participants**	55	56	110	58	22	52	50	59	107
**Total person-days = n (%)**	165 (100)	168 (100)	330 (100)	174 (100)	66 (100)	156 (100)	150 (100)	177 (100)	321 (100)
*Harvesting*	89 (54)	82 (49)	254 (77)	–	–	94 (60)	90 (60)	–	–
*Agrichemical (applicating/ mixing)*	–	13 (8)	59 (18)	–	66 (100)	62 (40)	60 (40)	39 (22)	–
*Supervising/Irrigating*	17 (10)	8 (5)	–	–	–	–	–	25 (14)	–
*Seed sowing*	–	54 (32)	–	–	–	–	–	–	–
*Crop maintenance*	8 (5)	–	–	–	–	–	–	57 (32)	–
*Driver/machine operator*	35 (21)	–	–	12 (7)	–	–	–	–	–
*Manual assistant*	–	–	–	111 (64)	–	–	–	–	–
*Machinery assistant*	–	–	–	14 (8)	–	–	–	–	–
*Road safety*	–	–	–	14 (8)	–	–	–	–	–
*Clay work and/or carrier*	–	–	–	–	–	–	–	–	228 (71)
*Oven work*	–	–	–	–	–	–	–	–	83 (26)
*Other mixed activities*	17 (10)	11 (6)	17 (5)	23 (13)	–	–	–	56 (32)	7 (3)

#### Baseline CKD prevalence

CKD status was determined for 568 participants. One participant in Round 1 died of ESKD before Round 2 and was classified as having CKD. A second participant with eGFR < 60 mL/min/1.73m^2^ at Round 1 emigrated from the region and was not counted in the prevalence estimate. Ten participants with eGFR < 60 mL/min/1.73m^2^ at Round 1 had eGFR > 60 mL/min/1.73m^2^ at Round 2 and were not considered as having CKD. Overall baseline prevalence of CKD was 7.4% (Table 3). MANOS investigators provided counseling and referrals for medical evaluation to participants based on their laboratory results.

**Table 3:** Participants with CKD Stages 3–5 at Baseline

CKD Stage (eGFR mL/min/1.73 m^**2**^)	n (%)
Overall CKD Prevalence, Stages 3–5 combined (< 60)	42 (7.4)
Stage 3a (45 to < 60)	9 (1.6)
Stage 3b (30 to < 45)	16 (2.8)
Stage 4 (15 to < 30)	9 (1.6)
Stage 5 (< 15)	7 (1.2)

Of participants with CKD, 16.6% (*n* = 7) had eGFR values below 15 ml/min/1.73min^2^ and were classified as stage 5 CKD (ESKD). Most participants determined to be CKD+ had stage 3 disease (59.5%, *n* = 25) (Table 3).

CKD prevalence increased with age. Among participants aged 18–24, fewer than 1% (0.6%) had CKD, whereas 5% of individuals aged 25–34 and almost 20% (18.9%) of those aged 35–45 had the disease (Table 4).

**Table 4:** CKD Cases, and Crude and Age-Standardized CKD Prevalence among MANOS participants by demographic variables

Demographic	CKD cases n (% of cases)	Crude Prevalence^a^ %	Age-standardized Prevalence^a^ % (95% CI)
**Age**			
18–24 (*n* = 172)	1 (2.4)	0.6	–
25–34 (*n* = 250)	14 (33.3)	5.6	–
35–45 (*n* = 147)	27 (64.3)	18.4	–
**Country**			
El Salvador (*n* = 279)	28 (66.6)	10.0	10.1 (6.7, 13.5)
Nicaragua (*n* = 290)	14 (33.3)	4.8	4.6 (2.3, 7.0)
**Family history**			
No father or brother with CKD (*n* = 457)	29 (69.0)	6.3	6.9 (4.5, 9.2)
Brother with CKD (*n* = 47)	6 (14.3)	12.8	9.1 (2.3, 15.8)
Father with CKD (*n* = 84)	10 (23.8)	11.9	9.4 (4.0, 14.9)
Father OR brother with CKD (*n* = 112)	13 (31.0)	11.6	9.0 (4.4, 13.6)
Father AND brother with CKD (*n* = 19)	3 (7.1)	15.8	10.8 (0.0, 22.1)
Father Unknown CKD status (*n* = 42)	3 (7.1)	7.1	9.2 (0.0, 18.5)
**Industry**			
El Salvador Sugarcane (*n* = 111)	16 (38.1)	14.4	14.1 (7.9, 20.3)
Nicaragua Sugarcane (*n* = 124)	1 (2.4)	0.8	0.6 (0.0, 1.8)
Brick (*n* = 107)	10 (23.8)	9.4	8.1 (3.3, 12.8)
Corn (*n* = 110)	10 (23.8)	9.1	11.6 (5.6, 17.6)
Plantain (*n* = 59)	3 (7.2)	5.1	4.9 (0.0, 10.2)
Road construction (*n* = 58)	2 (4.8)	3.4	2.9 (0.0, 6.7)

^a^ Denominator for prevalence appears in the first column of the corresponding row

Age-standardized prevalence among participants who reported a family member with CKD was higher compared to participants with no family history of the disease (Table 4). Among participants who reported neither a father nor a brother with CKD, age-standardized prevalence was 6.9% (4.5, 9.2%), while prevalence among participants who reported having a father *or* brother with CKD was 9.0% (4.4, 13.6%). Among 19 individuals who reported *both* a father and a brother with CKD, age standardized prevalence of CKD was 10.8% (0.0, 22.1%). Given small sample sizes, confidence intervals were wide.

Age-standardized prevalence of CKD differed across industries (Table 4). Prevalence was highest in Salvadoran sugarcane (14.1%), corn (11.6%), and brickmaking (8.1%). Nicaraguan sugarcane prevalence was less than 1%. This is not surprising as Nicaraguan sugarcane companies screen workers’ serum creatinine prior to the harvest season; employment is contingent on healthy kidney function. Excluding Nicaraguan sugarcane, the lowest prevalence was observed in road construction at nearly 3 %, followed by plantain at nearly 5 %.

#### Kidney function parameters

All kidney function parameters, except for albumin, differed between CKD+ and CKD- participants in age-adjusted regression models (Table 5).

**Table 5:** Estimated GFR among all MANOS participants and by CKD status at baseline with age-adjusted comparisons of serum kidney function parameters between groups at Round 1

Parameter	Reference range ^*a*^	All MANOS Mean ± SD	CKD+ Mean ± SD	CKD- Mean ± SD	Age-Adjusted Mean difference (95% CI)^***b***^
eGFR (ml/min/1.73min^2^) ^*c*^	> 90	106.7 ± 28.2	33.6 ± 15.5	112.7 ± 19.1	−68.6 (− 74.1, − 63.1)
Creatinine (mg/dL)	0.60–1.35	1.06 ± 0.81	3.10 ± 1.96	0.89 ± 0.21	2.07 (1.90, 2.26)
Albumin (g/dL)^*d*^	3.6–5.1	4.5 ± 0.26	4.4 ± 0.26	4.5 ± 0.26	0.03 (− 0.08, 0.13)
Calcium (mg/dL)	8.6–10.3	9.3 ± 0.38	9.1 ± 0.52	9.3 ± 0.36	− 0.17 (− 0.28, − 0.05)
Carbon dioxide (mmol/L)^*d*^	20–32	26.2 ± 3.0	25.4 ± 3.7	26.3 ± 3.0	− 0.96 (− 0.91, 1.00)^*e*^
Chloride (mmol/L)	98–110	100.7 ± 3.2	99.3 ± 5.2	100.8 ± 3.0	0.99 (0.98, 1.00)^*e*^
Creatine phosphokinase (U/L)^*f*^	39–308	241.8 ± 142.0	298.5 ± 162.4	239.1 ± 140.8	1.16 (0.92, 1.45)^*e*^
Glucose (mg/dL)	65–99	90.3 ± 16.0	96.17 ± 17.1	89.8 ± 15.8	3.61 (− 1.28, 8.50)
Blood urea nitrogen (BUN) (mg/dL)	7–25	12.3 ± 6.4	25.9 ± 9.7	10.8 ± 3.6	14.7 (12.8, 16.6)
Phosphorous (mg/dL)	2.5–4.5	3.6 ± 0.67	3.7 ± 0.92	3.6 ± 0.65	0.26 (0.06, 0.46)
Potassium (mmol/L)	3.5–5.3	3.9 ± 0.44	3.7 ± 0.66	4.0 ± 0.43	−0.19 (− 0.33, − 0.54)
Sodium (mmol/L)	135–146	138.0 ± 2.4	136.3 ± 3.5	138.1 ± 2.2	− 1.54 (− 2.26, − 0.82)
Uric acid (mg/dL)	2.5–8.0	5.8 ± 1.7	9.1 ± 2.2	5.6 ± 1.3	3.13 (2.70, 3.55)

^*a*^ Reference ranges are from Quest Diagnostics, with the exception of urea and total creatinine phosphokinase (CPK), which are from the Nicaraguan National Laboratory. Reference ranges are for men ages 20–49 years. The serum creatinine reference range for men ages 18–19 is 0.60–1.26 (mg/dL) and for serum calcium is 8.9–10.4 (mg/dL)

^*b*^ Age-adjusted mean difference by CKD status was calculated using linear regression

^*c*^ All values presented were collected prior to the work shift on Day 1 of baseline enrollment

^*d*^ Albumin and carbon dioxide were only assessed in samples from El Salvador (*n* = 279)

^*e*^ Log transformed for linear regression. Exponentiated beta coefficients and 95% CIs are displayed

^*f*^ Total creatinine phosphokinase was only assessed in samples from Nicaragua (*n* = 290)

Mean eGFR values at baseline among CKD+ participants were 33.6 min/ml/1.73 min2 (SD: 15.5) compared to 112.7 min/ml/1.73 min2 (SD: 19.1) among CKD- participants, while mean serum creatinine values were 3.12 mg/dL (SD: 1.96) and 0.89 (SD: 0.21), respectively. Serum calcium, potassium and sodium levels were lower among CKD+ participants compared to CKD- participants. Serum carbon dioxide, chloride, creatine phosphokinase, glucose, urea nitrogen, phosphorous, and uric acid levels were higher among CKD+ participants compared to CKD- participants. Notably, mean uric acid levels among CKD+ participants were 9.1 mg/dL (SD: 2.2) compared to 5.6 mg/dL (SD: 1.3), among CKD- participants, with an age adjusted mean difference of 3.13 mg/dL (CI: 2.70, 3.55).

## Discussion

Mesoamerican Nephropathy Occupational Study (MANOS) is a longitudinal cohort of agricultural and non-agricultural workers in two countries, El Salvador and Nicaragua. Given that traditional risk factors for CKD as well as prior diagnosis of the disease were exclusion criteria for recruitment – and kidney function screenings were required by some employers – the observed CKD prevalence of 7.4% in a working population of relatively young men is notable. For comparison, the United States’ CKD Surveillance System, coordinated by the US Centers for Disease Control and Prevention, estimates CKD among different sectors of the population based on national surveys. Analyses of one such survey by the Veteran’s Affairs health system estimated the prevalence of CKD stages 3–5, based on a single measure of eGFR < 60 mL/min/1.73m^2^, to be less than 1.6% among US veterans ages 40–49, and less than one half percent in veterans age 39 or younger [37]. Veterans in this dataset include individuals with hypertension, diabetes and known risk factors for CKD, criteria of exclusion for the MANOS study. A remarkably high percentage of otherwise healthy young men in MANOS had low kidney function at baseline.

Our results are similar to community prevalence studies in areas of Nicaragua and El Salvador considered high CKDu risk [38–40]. In rural Northwest Nicaragua, O’Donnell et al. estimated a CKD prevalence of 7.8% based on eGFR < 60 mL/min/1.73m^2^ among men ages 18 to 42 [38]. However, as with the previously mentioned data on veterans in the US, neither prior CKD diagnosis nor CKD risk factors were criteria for exclusion and prevalence was calculated based on eGFR at one time-point [38]. Gonzalez-Quiroz et al. estimated CKD prevalence of 9.5% among men ages 18–30 in nine communities in Northwestern Nicaragua, similarly based on a single time point eGFR < 60 mL/min/1.73m^2^ [39]. Exclusion criteria were similar to MANOS, indicating an extraordinarily high prevalence of undetected CKD not associated with classic risk factors of diabetes or hypertension [39]. In El Salvador, a community study of five localities estimated an overall prevalence of 6.6% among men ages 20–60 years based on a single eGFR < 60 mL/min/1.73m^2^ [40]. However, among coastal sugarcane communities in the study prevalence was 13.2 and 15.0%, respectively [40]. Ten MANOS participants would have been considered CKD cases if we had relied only on the first round of data collection. Their eGFR < 60 mL/min/1.73m^2^ at Round 1, and went above 60 at Round 2. It is possible that their kidney function recovered after the harvest season; and that had we collected Round 2 data a full year later, their eGFR would have been low again. It is possible our prevalence is an underestimate of CKDu, and/or that we are observing AKI events. Analyses of the long-term data will enable us to evaluate fluctuations by season and overall trajectory.

Differences in age-standardized prevalence between industries in the MANOS cohort was noteworthy. Salvadoran sugarcane workers and corn workers had the highest prevalence of disease – greater than 10% – with brickmakers also elevated at 8.1%. While several studies have included sugarcane workers in El Salvador, this is the first to include workers in corn who, for many years, have been sounding the alarm regarding CKDu in their communities [29]. Nicaraguan sugarcane workers were the lowest at less than 1% followed by road construction and plantain at 2.9 and 4.9%, respectively. As previously mentioned, screening procedures in Nicaraguan sugarcane likely, at least partially, explain this low prevalence. However, we also note the diversity of job tasks performed among sugarcane workers in El Salvador compared with sugarcane workers in Nicaragua who exclusively harvested cane or worked with agrichemicals in Round 1.

It is also notable that a non-agricultural industry, artisanal brickmaking, had the highest prevalence in Nicaragua. We observed a 9.4% unadjusted prevalence in brickmakers, indicative of increased risk of CKD in this population. This finding is consistent with a prior study by our team among brickmakers in which we estimated a CKD prevalence of 14.1% among men [11]. Contributors to the higher prevalence in the prior study likely include the fact that, unlike in MANOS, we did not exclude individuals older than age 45, nor workers with a known diagnosis of CKD or CKD risk factors [11].

Across all industries in MANOS, we observed higher prevalence of CKD among participants with a family history of the disease. Similar associations have been found in other studies [41, 42], including our prior study in brickmakers where the prevalence of CKD was twice as high in those with a brother or father with CKD [11]. Association with family history could represent shared environmental exposures, genetic factors, or a combination of the two.

Naturally, and by definition, CKD+ participants had eGFR values that were markedly impaired compared to CKD- participants. However, two biochemical parameters stand out as particular to CKDu. First, we observe relative hypokalemia; the potassium level in CKD+ participants is lower than CKD- participants. In most cases of CKD the serum potassium would likely be considerably elevated, however other studies have indicated hypokalemia is common in CKDu [16, 43]. Future analyses should examine hypo- and hyperkalemia by stage of disease. Evidence suggests a tubular abnormality in potassium handling that may provide protection against hyperkalemia; a dangerous consequences of kidney failure. We also observed an elevation of serum uric acid in CKD+ participants that appears out of proportion to the degree of GFR loss, consistent with previous reports [44]. These electrolyte abnormalities are also consistent with biopsy series that demonstrate primarily tubulointerstitial injury [45]. It remains to be seen whether these biochemical abnormalities precede or follow GFR loss, if they are clues to the cause of the disease, and whether they can serve as useful biomarkers for early detection of CKDu and differentiation from other forms of kidney disease.

By following the MANOS cohort longitudinally, we will examine associations between occupational exposures and decline in kidney function. Subsequent rounds of data collection with MANOS participants will enable us to estimate both CKD incidence and the rate of decline in kidney function as measured by eGFR, among workers in both countries and across industries. Future analyses include measuring urinary biomarkers of kidney injury at baseline, assessing occupational history for each worker (duration and job tasks), and examining associations with kidney injury at baseline and kidney function over time. Exposure to heavy metals, heat stress and agrichemicals will be examined individually and in combination as possible predictors of the aforementioned kidney outcomes.

### Challenges and limitations

It was not possible to collect all of the baseline data during the dry season, and the final participants recruited in some industries were monitored during the beginning of the rainy season (May 2018). Ambient temperatures are different in the rainy season as compared to the dry season and many industries either reduce work intensity or alter job tasks during the rainy season.

Political instability and gang-related violence at times posed a challenge for baseline participant monitoring. Political instability in Nicaragua toward the end of baseline data collection resulted in uncertainty and confusion in the work environment. Companies changed workers’ schedules and individual workers were not always able to reach their place of employment due to the lack of a functioning transportation system, making it difficult for the MANOS team to characterize three consecutive workdays for each worker. At times, the MANOS research team had to pause fieldwork until tensions abated and movement was possible. In Nicaragua, the team carried a small freezer in their vehicle to mitigate concerns related to the unpredictable presence of blockaded roads increasing the amount of time samples were in transport. In El Salvador, gang-related violence required the accompaniment of guards to certain worksites and very well-planned logistics. Some individuals had other obligations during normal work hours and arrived to work very early in the morning, hours before the start of the traditional work-shift, to work a full-day before having to depart. Political and social instability in Nicaragua also forced us to re-evaluate laboratory analyses for specimens collected from Round 1, shifting analyses for El Salvador samples out of the Nicaragua state laboratory to Quest Diagnostics in Marlborough, MA, USA. This change resulted in some differences in which kidney function parameters were assessed for all participants, due to different capacities at each laboratory. With all future analyses conducted at Quest using standardized technologies, these concerns have been minimized in future rounds.

Finally, the prevalence of CKD is measured by serum creatinine to estimate GFR. Relying solely on this measure of disease to estimate prevalence at baseline could be considered a limitation.

## Conclusions

Despite efforts to recruit healthy workers, over 7% of our participants had CKD at baseline, indicating notable hidden kidney disease within this working population. The highest baseline prevalence was observed among Salvadoran sugarcane workers, over 14%. We also observed high CKD prevalence in the non-agricultural industry of brickmakers, adding to evidence that non-agricultural exposures are associated with kidney disease. The MANOS cohort provides us with a unique opportunity to study occupational and environmental exposures, as well as genetics, that are hypothesized causal factors in the development and/or progression of MeN in Central America. Participant recruitment from two countries and five industries increases our ability to study and understand the breadth of the epidemic.

## Data Availability

The datasets analyzed for the current study are included in this published article as a supplementary file. Details regarding the equipment and protocol for exposure monitoring are also included as a supplementary file.

## Abbreviations

AGDYSA: Agency for Agricultural Development and Health
AKI: Acute kidney injury
BUMC: Boston University Medical Campus
CKD: Chronic kidney disease
CKDu: Chronic kidney disease of unknown etiology
CKD-EPI: Chronic Kidney Disease Epidemiology Collaboration
CNDR: Nicaragua Ministry of Health National Laboratories
eGFR: Estimated glomerular filtration rate
GFR: Glomerular filtration rate
KDIGO: Kidney Disease Improving Global Outcomes consortium
MANOS: Mesoamerican Nephropathy Occupational Study
MDRD: Modification of Diet in Renal Disease Study
MeN: Mesoamerican Nephropathy
PPE: Personal protective equipment
T_c_: Core body temperature
WBGT: Wet Bulb Globe Thermometers
